# Study on Influencing Factors of Hydraulic Engineered Cementitious Composites Layer Bonding Performance

**DOI:** 10.3390/ma16206693

**Published:** 2023-10-14

**Authors:** Yupu Wang, Jiazheng Li, Yan Shi

**Affiliations:** 1 Institute of Materials and Structure, Changjiang River Scientific Research Institute, Wuhan 430010, China; wangyupu29@126.com (Y.W.); 18163559730@163.com (Y.S.); 2 Research Center of Water Engineering Safety and Disaster Prevention of Ministry of Water Resources, Wuhan 430010, China

**Keywords:** hydraulic engineered cementitious composites, normal mortar, layer, flexural strength

## Abstract

The layer bonding performance of hydraulic engineered cementitious composites (HECCs) plays an important role in their application in hydraulic buildings. This performance encompasses the bonding between layers of HECCs, as well as between HECCs and normal mortar (NM) layers. The influence of various factors on the layer bonding performance of HECCs was investigated. These factors included different pouring intervals (0 min, 20 min, 40 min, 60 min, 2.5 h, 7 days, 14 days, and 28 days), pouring directions (horizontal and vertical), degree of saturation (100%, 70%, 50%, 30%, and 0%), and surface roughness (varying sand-pour roughness). It was found that longer pouring interval times led to a decrease in the layer bonding performance, and the strength of the layer bonding fell below 50% compared to concrete without layers, with the lowest recorded strength being only 1.12 MPa. The layer’s horizontal flexural strength surpassed the vertical flexural strength, but the horizontal compressive strength fell below the vertical compressive strength. Additionally, the bonding performance of the substrate at 0% saturation was 15–20% lower compared to other saturation levels. Notably, roughness significantly enhanced the performance of HECC layers, with improvements reaching a maximum of 180–200%. Furthermore, the layer performance of HECCs and NM experienced an improvement of 20.5–37.5%.

## 1. Introduction

Concrete is one of the most extensively utilized building materials globally, and current research has continuously enriched the material composition and performance of concrete [1,2,3,4,5,6,7]. In the 1990s, Prof. Li of the University of Michigan developed ultra-high-toughness cementitious composites with tensile strain hardening characteristics and high toughness by using a micro-mechanics-based performance-driven design method. Li et al. gave the definition of the name “Engineered Cementitious Composites”, abbreviated as ECCs, which have good ductility, multiple microcracks appearing on the ECC specimens under uniaxial tensile loading with the crack width controlled at 100 μm, and a tensile strain capacity of usually more than 2% [8,9,10]. ECCs have strong fatigue resistance, strong deformation ability, and good durability and crack resistance, which makes them widely used in engineering, such as for jointless pavements [11], structural seismic resistance [12], and bridge deck connection plates [13].

In response to the actual situation of water conservancy projects, the Changjiang River Scientific Research Institute of Changjiang Water Resources Commission proposed the concept of ECCs applicable to hydraulic construction (hydraulic engineered cementitious composites, HECCs), which was successfully applied for the first time in the localized part of a hydropower station corridor. Compared to ECC materials, HECCs can adopt a wider range of raw material varieties, such as engineered local sand and gravel used to prepare the required fine aggregates. Moreover, the maximum particle size can be increased to 1.25 mm, the 28-day compressive strength is C25–C40, the 28-day elastic modulus is less than 20 GPa, and the elongation rate is between 1% and 3% [14]. However, there are fewer reports on HECCs in domestic hydraulic buildings. Li [14] combined the characteristics of HECCs and proposed the concept of their application in hydraulic buildings. In the locations of a rockfill dam foundation corridor, panel rockfill dam seepage control panels, the clay heart wall of a rockfill dam, an upstream seepage control structure of a milled concrete dam, and the foundation constraint zone of an arch dam, the original concrete was replaced by a reasonable arrangement of HECCs to improve the crack resistance and seepage control ability of the whole hydraulic building. The various applications of HECCs in different hydraulic structures involve the issue of layer bonding performance, which directly affects the effectiveness of HECCs. Therefore, this paper explores the influencing factors of HECC layer bonding performance, aiming to provide guidance for water conservancy engineering practice.

Concrete layers tend to have weak bonding performance. The bonding effect produces a layer of water film on the concrete layer, the water–cement ratio of the new concrete layer becomes higher, and, at the same time, the crystallization of caliche and hydroxide caliche on the layer increases, which hinders contact between the new and old concrete and thus reduces the strength of the layer [15]. Gao et al. [16] concluded that it is difficult for the new and old concrete to hydrate and fuse with each other. There may be an extrusion problem of aggregate, which prevents the cement paste at the layer from penetrating into the pores of the old concrete, thus causing the bond transition region in the new concrete to be not tight enough. It has also been suggested that the interface between old and new concrete is similar to the interface of aggregate and cement bonding and that there is a wall effect between the old and new concrete layers, resulting in the appearance of weak surfaces forming transition zones [17]. Fan et al. [18] and Santos et al. [19] investigated the bonding properties of old and new plain concrete layers, and Wang et al. [20] studied the bonding properties of ECCs with ordinary concrete. Diab et al. [21] studied the bonding properties of self-compacting concrete layers, and Farzad et al. [22] studied the bonding properties of UHPC to ordinary concrete layers. There are many factors affecting the layer bonding properties. Qin et al. [23] and Qian et al. [24] studied the effect of layer interval time on the layer properties based on mechanical strength, microhardness, and chloride ion penetration, and all demonstrated that the layer bonding properties were weakened by increasing the layer interval time. Zega et al. [25] investigated the effect of the pouring direction on the layer properties. Beushausen et al. [26] and Kamada et al. [27] investigated the effect of surface moisture on the layer properties, but the experimental results were discrete, and the relationship between wet and dry substrate layer properties was not clear. Roughness significantly increases the dimension bond strength, and higher substrate roughness ensures a larger contact area between the two types of concrete, resulting in a better bond, as evidenced by the results of Zhang et al. [28] and Tian et al. [29].

HECCs are currently in the early stages of practical application, with limited research conducted on their layer-related issues. This study aims to address this research gap by investigating the factors influencing the bonding performance between HECC layers and HECC-NM layers. The investigation will focus on the following aspects: (1) the variation patterns of layer flexural strength at different pouring intervals, (2) the impact of pouring directions (horizontal and vertical) on both compressive and flexural strength of the layers, (3) the performance variations of the layers under different substrate saturation conditions, and (4) the influence of surface roughness (varying sand-pour roughness). This research can offer practical guidance for the further advancement and promotion of HECCs in water conservancy projects.

## 2. Materials and Methods

### 2.1. Experimental Raw Materials and Properties

The materials used in the experiment included PO42.5-grade cement, class F fly ash, artificial sand, superplasticizer (SP), hydroxypropyl methyl cellulose-based viscosity modifying admixture (VMA), and polyvinyl alcohol (PVA) fiber. SP was used to reduce water consumption by nearly 40%, VMA was used to increase the consistency, and PVA fibers were used to improve the ductility of the cement substrate. The cement was normal Portland cement produced by Sichuan Esheng Cement Company (Emeishan City, China), and the basic properties of the cement are shown in Table 1, which meet the relevant technical requirements of GB/T 175-2020 [30] for 42.5 normal Portland cement. Fly ash was the Jintang Class I fly ash mineral admixture, and the test results of basic properties are shown in Table 2. The test results show that the test indexes of fly ash satisfy the technical requirements of class F Class I fly ash in DL/T 5055-2007 [31]. The chemical composition of the cement and fly ash is shown in Table 3. For fiber, as the most important toughening material in HECCs, the test selected Anhui Wanwei’s short-cut PVA fiber products, and the quality indicators provided by the manufacturer are shown in Table 4. The sand used was the artificial sand sampled at the project site, with a maximum particle size of 1.25 mm for the black mica quartz schist, and the distribution of artificial sand particles is shown in Table 5. The test results of concrete properties of the SP tested are shown in Table 6, which meet the relevant technical requirements of DL/T 5100-2014 [32].

### 2.2. Material Mixing Ratio

The HECC used in the experiment adopted a water/binder ratio (w/b) of 0.33, fly ash dosage of 50%, and fiber volume dosage of 2%. In order to ensure the fluidity of the mixture, the unit water consumption was selected as 320 kg/m^3^, the SP dosage was 0.8%, the VMA was 0.05%, and the experimental measured jumping table fluidity was 165 mm. The NM adopted a w/b of 0.33 and a fly ash dosage of 50%. In order to ensure similar fluidity with the HECC, the unit water consumption was selected as 270 kg/m^3^, the SP dosage was 0.8%, and the experimentally measured table hopping fluidity was 160 mm. The specific mixing ratios are shown in Table 7.

### 2.3. Test Methods

#### 2.3.1. HECC Mixing Process

**Figure 1:**
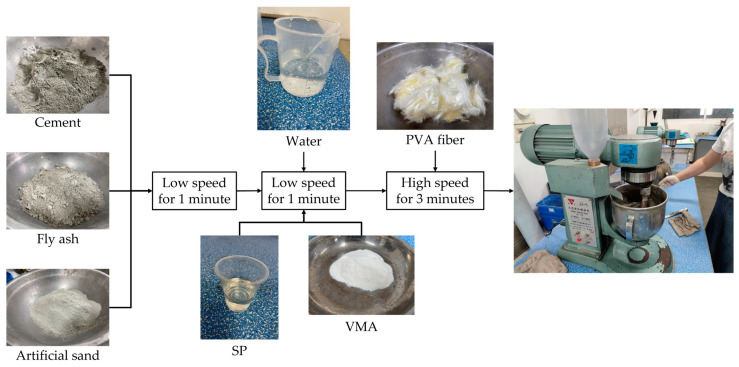
HECC Mixing process.

The degree of uniformity of fiber dispersion has a greater impact on the performance of HECCs, and the mixing system is a key factor affecting the fiber dispersion effect. Wuxi Jianyi JJ-5 cement mortar mixer (Wuxi, China) was used in the experiment, and the whole mixing process lasted 6–8 min. After weighing the corresponding components according to the ratio, the cement, fly ash, and artificial sand were firstly mixed dry at low speed for 1 min, the SP and VMA were mixed with homogeneous water at low speed for 2 min, and, finally, the PVA fibers were added to continue the mixing process at high speed for 3–5 min. After stirring, the HECC material was loaded into the test mold, and the mold was removed after 24 h of curing in the curing box and then moved to the standard curing room for curing for 7 days or 28 days for testing. The mixing process is shown in Figure 1.

#### 2.3.2. Preparation of Specimens

**Figure 2:**
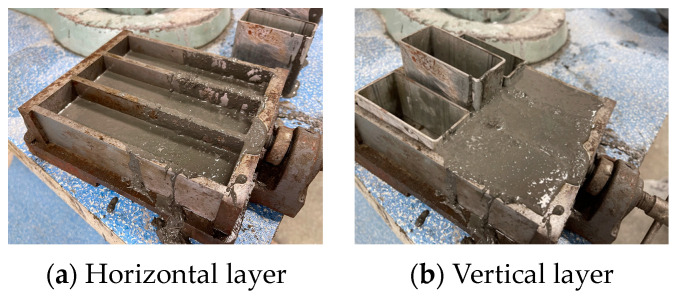
Layer formation diagram.

Different from the one-time pouring of the whole specimen, the layer specimen needs to be molded two times. In order to facilitate the description, the first poring layer is the substrate layer, and the later poring layer is the overlay layer. When preparing the specimens, the size of a single specimen was 40 mm × 40 mm × 160 mm. When molding the horizontal layer, a 20 mm thick substrate layer was poured, and then a custom-made steel mold was used to layer the top. When forming the vertical layer, a steel mold half the length of the specimen was placed in advance, and then the substrate layer was poured. Then, combined with the experimental needs, we took away the steel mold and poured the other half of the overlay layer at certain intervals. The layer formation diagram is shown in Figure 2.

#### 2.3.3. Layer Bonding Performance Test Method

**Figure 3:**
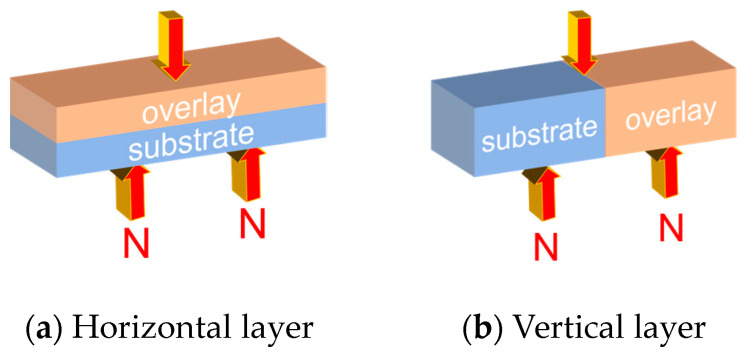
The layer flexural strength test diagram.

**Figure 4:**
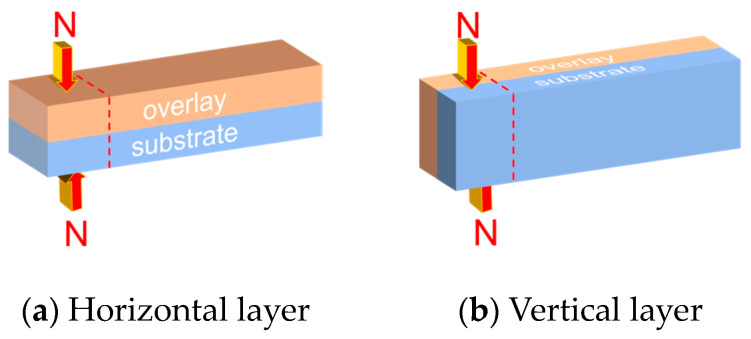
The layer compressive strength test diagram.

**Figure 5:**
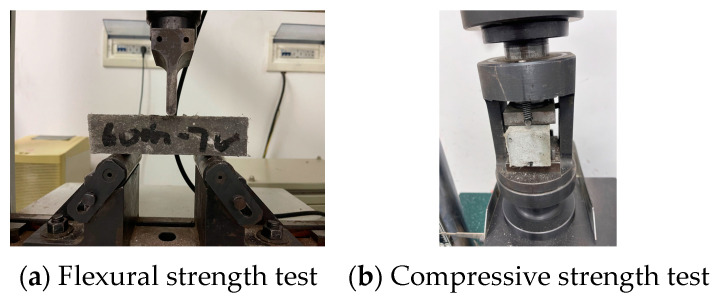
The flexural strength and compressive strength test device and layout.

All specimens were tested by using the method of GB/T 17671-2021 [33] for compressive strength and flexural strength determination, for which the flexural strength was divided into the horizontal layer and vertical layer, and the specimens were placed and loaded as shown in Figure 3. The compressive strength was divided into horizontal and vertical layers, and the test specimens were placed and loaded as shown in Figure 4. Figure 5 shows the device and layout during the actual test. The arrow direction in the figure represents the direction of load (pressure), and the dashed line represents the range of compression. (If the pouring direction and strength are not mentioned in the following experiments, the flexural strength of the vertical layer is always the default).

#### 2.3.4. Quantitative Analysis of Substrate Saturation

**Figure 6:**
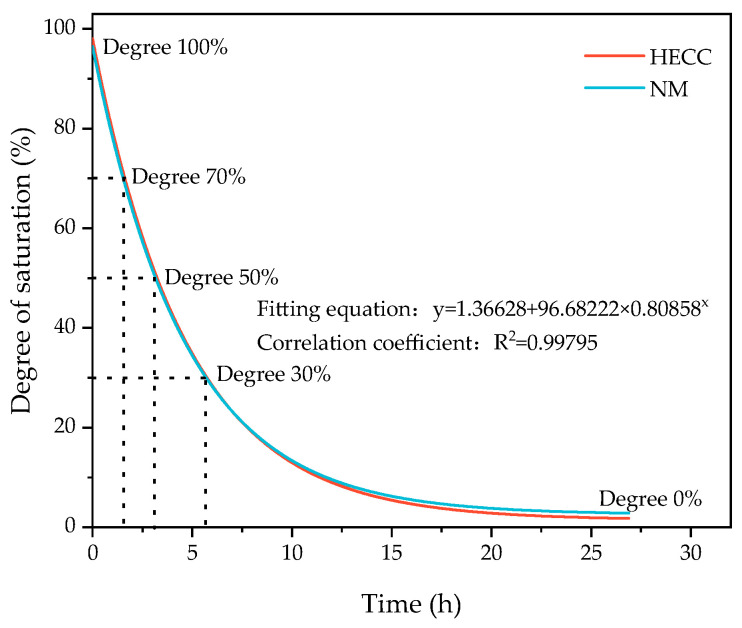
Curves of HECC and NM saturation changing with time in 105 °C oven.

For quantitative calculation of substrate saturation [34], the specimens were maintained dry corresponding to the age of the surface water. The process involved weighing the specimen water-saturated mass *m_w_*. Then, the specimen was placed in the oven at 105 °C to dry, taken out to weigh the mass of *m_i_* at certain intervals until the mass no longer changed, and ultimately dried to a constant weight of the mass of *m_d_*. Substrate saturation *S* corresponding to the different drying times was calculated according to Equation (1), and the corresponding relationship between drying time and substrate saturation is shown in Figure 6. The overall trend of HECCs and NM was close to the same fitting formula and achieved a good fitting effect.

(1) $$S=\frac{m_{i}-m_{d}}{m_{w}-m_{d}}\times 100\%$$

#### 2.3.5. Surface Roughness Creation Methods and Measurement Methods

**Figure 7:**
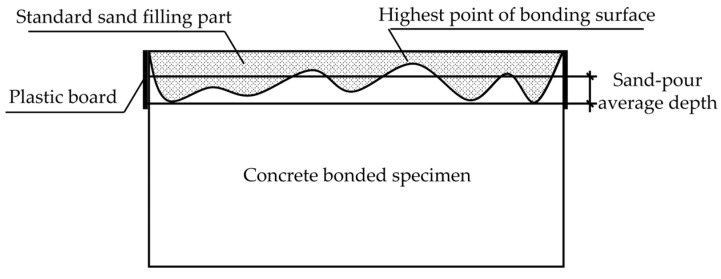
Sand-pour method to test the roughness of the bonding surface schematic.

**Figure 8:**
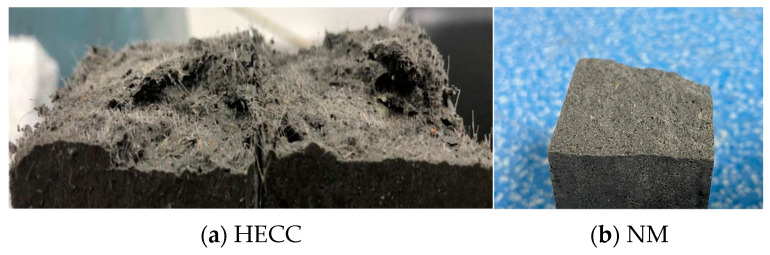
Typical fracture surface.

**Figure 9:**
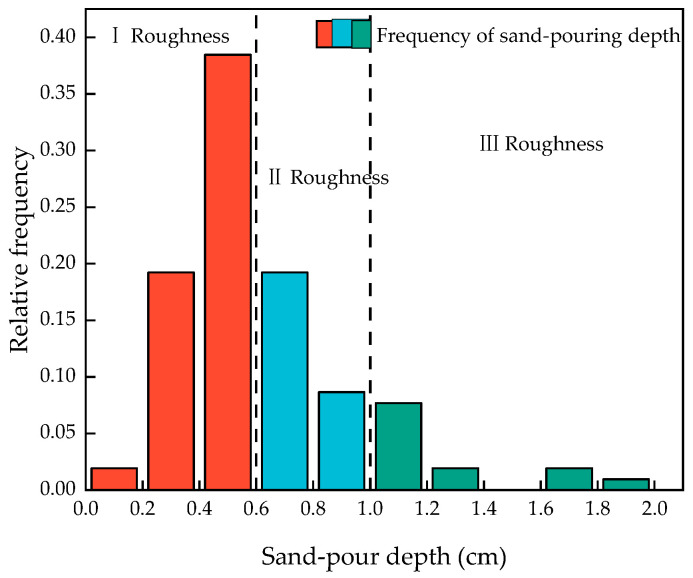
HECC frequency of sand-pour depth.

Traditional surface roughness creation methods include sandblasting, chiseling, high-speed water rushing, etc. [35]. These types of methods are often for the treatment of the concrete surface, and, at the same time, the treatment area is large. The specimen used in this experiment was a square with a cross-sectional area of 40 mm × 40 mm, which was a smaller size compared to the complete specimen. In order to better simulate the actual situation, the flexural test was carried out on the complete specimen. We put one part after fracture to two parts of the substrate layer into the mold and poured the other half of the layer for subsequent experiments. For the quantitative characterization of roughness, the experiments used the traditional sand-filling method [36], and the process is as follows: as shown in Figure 7, four pieces of a plastic plate surrounded the concrete bonding surface so that the top surface of the plastic plate and the highest point of the convex part of the bonding surface was flush, to which the standard sand was filled to exceed the bonding surface. The top surface of the plastic plate was smoothed until no more sand particles fell, the test piece of the bonding surface of the sand was poured into the cylinder, and its volume was measured and repeated. We poured all of the sand on the bonding surface of the test piece into the measuring cylinder and measured its volume. We repeated the above procedure three times and recorded the measurement results. The roughness of the bonding surface could be calculated by using the sand-pour average depth according to Equation (2). After the fracture test of the HECC, the fracture surface was not flat due to the ductility of HECC itself as shown in Figure 8a, whereas NM was brittle and the fracture surface was relatively flat as shown in Figure 8b. By measuring the surface roughness of the HECC fracture specimens, the distribution frequency of the sand-pour depth was statistically determined as shown in Figure 9, and based on the distribution frequency, 0–0.6 cm was defined as Class I roughness, 0.6–1.0 cm was defined as Class II roughness, and more than 1.0 cm was Class III roughness. The measurement of the surface roughness of NM revealed that the sand-pour depth was basically around 0.2 cm.

(2) $$\mathrm{Sand}-\mathrm{pour}\;\mathrm{average}\;\mathrm{depth}=\frac{\mathrm{Standard}\;\mathrm{sand}\;\mathrm{volume}}{\mathrm{Section}\;\mathrm{area}\;\mathrm{of}\;\mathrm{bonding}\;\mathrm{surface}}$$

## 3. Results and Discussion

### 3.1. Basic Mechanical Properties of Materials

**Figure 10:**
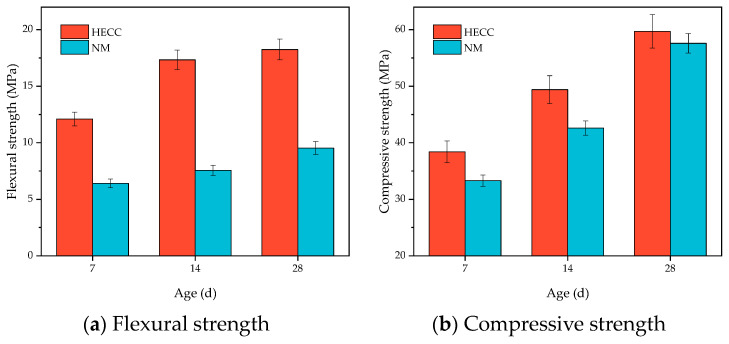
HECC and NM’s basic mechanical properties.

**Figure 11:**
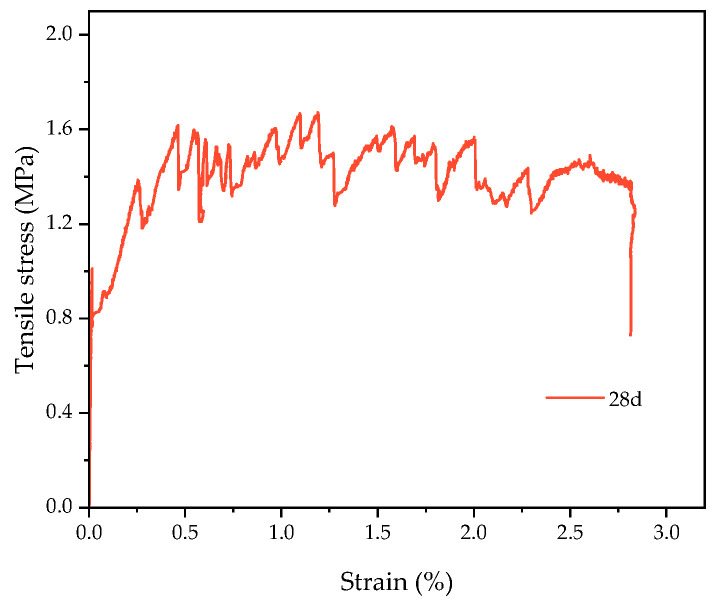
HECC stress–strain curve under tensile load.

The basic flexural mechanical properties of the HECC and NM are shown in Figure 10a. With the increase in age, the flexural strength of both the HECC and NM increased, the flexural strength of the HECC was significantly higher than that of NM, and the flexural strength of the HECC reached 18.25 MPa and that of NM reached 9.54 MPa at the age of 28 days. The basic compressive mechanical properties of the HECC and NM are shown in Figure 10b. It shows that the trend is consistent with the flexural strength. At the age of 28 days, this gap was smaller; this gap became smaller with the increase in age. The HECC reached 59.7 MPa, and NM reached 57.6 MPa. The main contributing factor is that the hydration of cement continues, and the strength also increases with age. Due to the bridging effect of the fiber, the material can bear a greater load when it is loaded, making the flexural strength higher than that of ordinary mortar. The compressive strength is directly related to the w/b ratio, and the w/b ratio of the HECC and NM is the same, so the compressive strength is relatively close. The HECC possesses the characteristics of better strain hardening, and under a tensile load, it exhibits the characteristics of multiple microcracks. With reference to the tensile properties of JC/T 2461-2018 [37], we obtained the tensile stress–strain curves of HECC at the age of 28 days. As shown in Figure 11, the maximum tensile stress of 1.67 MPa corresponds to a strain of 1.2%, which shows good ductility.

### 3.2. Study of Factors Influencing Layers

#### 3.2.1. Influence of Pouring Interval on the Performance of Layers

**Figure 12:**
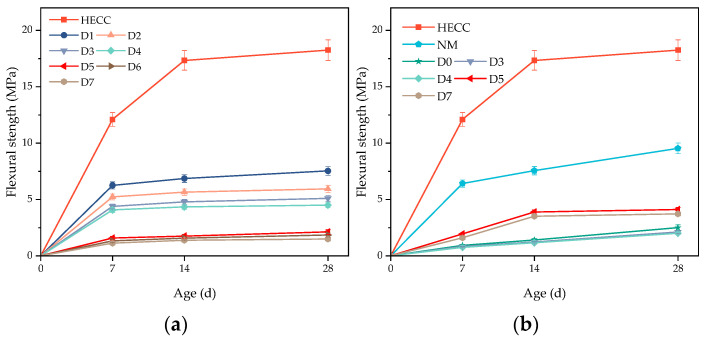
Influence of different pouring interval times on layer bonding properties. (**a**) Layer performance of HECC and HECC layers; (**b**) Layer performance of HECC and NM.

The HECC and NM were taken as the substrate layer, and the other half of the HECC was poured after a certain time interval. The numbering of different pouring intervals and the corresponding meanings are shown in Table 8. From Figure 12a, it can be seen that as long as there was an interval time between pouring, the specimens were not molded in one go, and the flexural strength of all specimens at the layer of the HECC and the layer between the HECCs was lower than that of the specimens molded in one go. The overall pattern is that the flexural strength of the layer decreased with the increase in the interval time between pouring. At shorter intervals of 20 min, 40 min, 60 min, and 2.5 h, the flexural strength of the layers was close to each other but lower than 50% of the strength of the specimens poured as a whole, with a minimum of 4.07 MPa. After the hardening of the substrate layer, the flexural strength of the layers was further reduced significantly, with the flexural strength of the layers being close to each other at intervals of 7 days, 14 days, and 28 days and reaching a minimum of 1.12 MPa at 28 days. From Figure 12b, it can be seen that even without pouring intervals, the maximum HECC and NM layer flexural strength was only 2.5 MPa after 28 days of curing, but the experiment observed that the layer flexural strength before the NM substrate hardened was less than the layer flexural strength after the NM substrate hardened, which is exhibited by the HECC being different from the HECC layers. After hardening, the flexural strength of the layers could reach up to 4.1 MPa, which was still smaller than that of the NM specimens with no layers. Despite the lower flexural strengths of all the layers, with the curing of the layer specimens, they still showed a small increase in strength with the increase in the age of curing.

The surface of the substrate did not harden before the 2.5 h pouring interval, and as long as a certain pouring interval existed, a water film existed on the surface of the substrate and became progressively thicker with the increase in the interval, thus hindering the bonding between the layers [38]. Meanwhile, an HECC molded in a single run without pouring intervals, due to the uniformly dispersed PVA fibers, can provide enough bridging stress, which inhibits the further expansion of the crack width after cracking. At the same time, it assumes the stress released by the substrate and relies on the interfacial bonding to transfer the stress to the surrounding un-cracked substrate and then produce new cracks, resulting in high flexural strength [39]. Once there is a pouring interval, there is no fiber penetration at the layer, and the bridging ability of the fibers cannot be exerted, which makes the vertical flexural strength of the layer only half of that of the layer without the layer and close to the NM strength in the same proportion even with a short interval.

When the substrate has hardened, the interfacial bond mainly consists of mechanical, van der Waals, and chemical forces according to the layer bonding mechanism of old and new concrete. The hardened substrate layer force mainly includes mechanical and chemical forces, and the mechanical occlusion force formed on the respective surfaces by the radial growth of cement hydration products in the substrate and the cover layer plays a major role. When the substrate age reaches 28 days, the cement hydration of the substrate is basically completed, the cement hydration reaction rate is slowed down, and the layer vertical flexural strength is lower. If the new concrete is poured before the hydration of the old concrete is completed, a larger interfacial bond is formed. Fan et al. [18] found that the splitting strength of the old and new concrete decreases with the age of the old concrete. When the age of the old concrete reaches 10 days, the splitting tensile strength decreases by about 20–30% compared to that at day 1, and, after that, the decrease tends to slow down with the increase in age.

#### 3.2.2. Influence of Pouring Direction on Layer Performance

**Figure 13:**
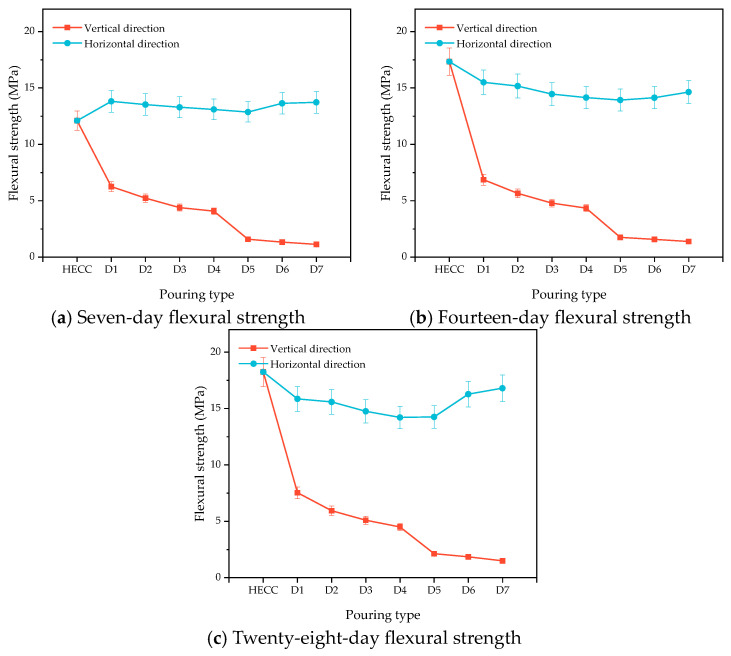
Influence of different pouring directions on the flexural strength of HECC and HECC layers.

**Figure 14:**
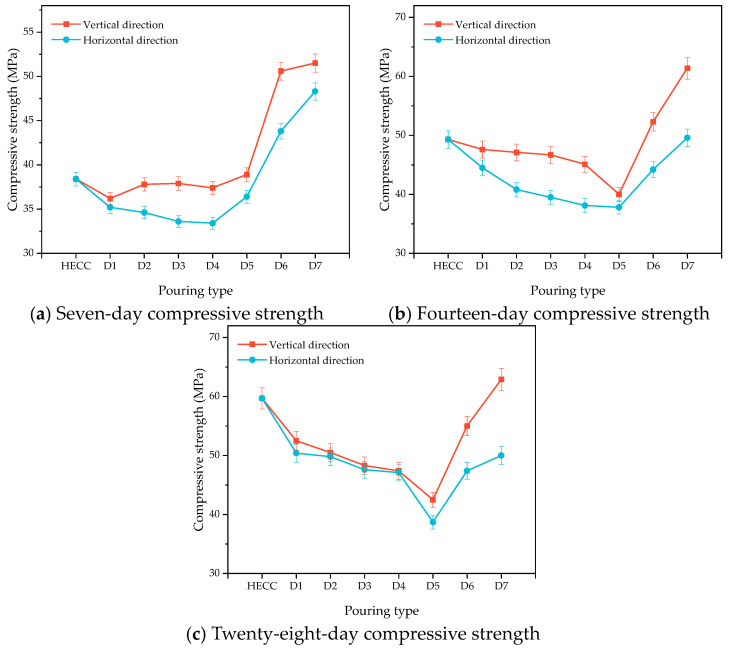
Influence of different pouring directions on the compressive strength of HECC and HECC layers.

**Figure 15:**
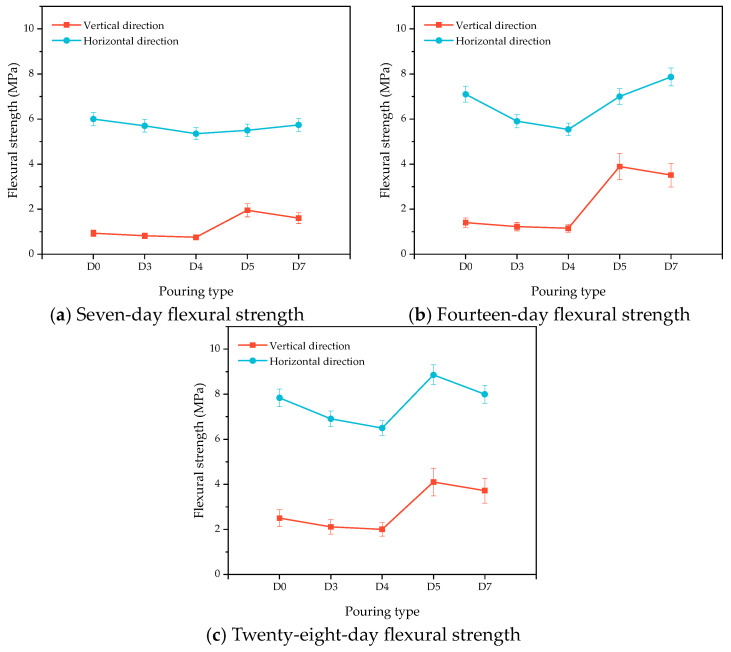
Influence of different pouring directions on the flexural strength of HECC and NM layers.

**Figure 16:**
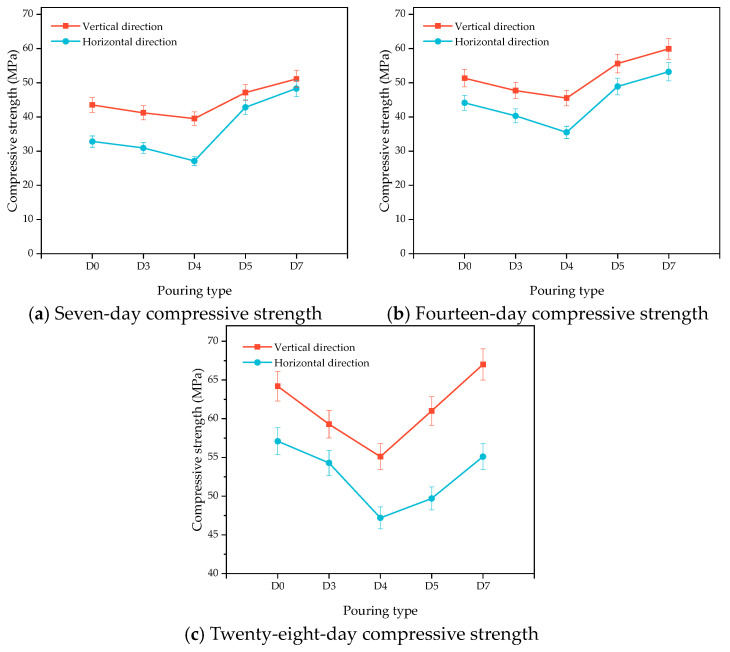
Influence of different pouring directions on the compressive strength of HECC and NM layers.

Figure 13a–c show the changes in the flexural strength of the HECC and HECC layers after 7 days, 14 days, and 28 days of curing. It can be seen that at all ages, the flexural strength in the horizontal direction is higher than that in the vertical direction, and the flexural strength of the vertical layer decreases gradually with the increase in the interval time, with the lowest being 1.12 MPa, 1.38 MPa, and 1.49 MPa at different ages. In contrast, the horizontal layer exhibits a tendency of decreasing and then increasing, and, at the same time, the change amplitude is small; even at the age of 7 days, it exhibits a higher flexural strength than that of no layer. Figure 14a–c show the variation in compressive strength in turn. Unlike the carryover folding strength, the compressive strength of the vertical layer is higher than that of the horizontal layer, and all of them show a decreasing and then increasing trend up to 62.9 MPa, which is higher than that of the compressive strength of the no-floor HECC specimen. Figure 15a–c show the variation in the flexural strength between the HECC and NM layers, and the flexural strength of the horizontal layer is also higher than that of the vertical layer, which is higher than that of the no-floor layer. The flexural strength of the horizontal layers is also higher than that of the vertical layers. Figure 16a–c shows the variation in compressive strength between HECC and NM layers. The compressive strength of the vertical layers is higher than that of the horizontal layers, decreasing and then increasing with the increase in interval time, and the compressive strength of the layers reaches a maximum of 67 MPa at the 28-day pouring interval time, which is higher than that of the layers at no interval time.

For the flexural strength of the layers, the strength of the bonded specimens cast in the horizontal direction is higher than that of the specimens cast in the vertical direction. The reason for this difference is that, as Wang et al. [20] concluded, when pouring horizontally in the molding direction, the HECC sinks and the bubbles near the interface rise, creating closer contact between the two and resulting in higher flexural strength. Vertical pouring causes an uneven distribution of bond strength along the height direction, and it is easy to form holes and a segregated water layer under the protruding part of the bonding surface, thus causing low bond strength. Meanwhile, the horizontal layer is perpendicular to the loading direction in the flexural test, resulting in the loading; the layer direction is not directly subjected to the action of the load; and, at the same time, there may be friction between layers, further increasing the flexural strength. In contrast, when the vertical layer is parallel to the loading direction, the layer serves as the weakest surface when loading and has lower flexural strength. Zega et al. [25] also observed a similar phenomenon. As for the phenomenon of compressive strength, that is, when the vertical layer compressive strength is higher than that of the horizontal layer, we analyzed the possible reasons for the compressive strength test according to the compression specimen deformation and the point at which the peak load stops growing and begins to decline. The horizontal layer of the specimen, between its two layers, shows the existence of a certain difference in the compressive strength. The test stops when one side of the side produces a large deformation. This is judged as the compressive strength, so it is small. The vertical layer combines two parts of the layer as they share a common surface load, and the strength is correspondingly high. This gap, with the age of the gap, becomes bigger and bigger. However, the results also show that the existence of the layer has little effect on the compressive strength of the layer, which is even higher than the overall compressive strength of the specimen without any interval time.

#### 3.2.3. Influence of Substrate Saturation on Layer Properties

**Figure 17:**
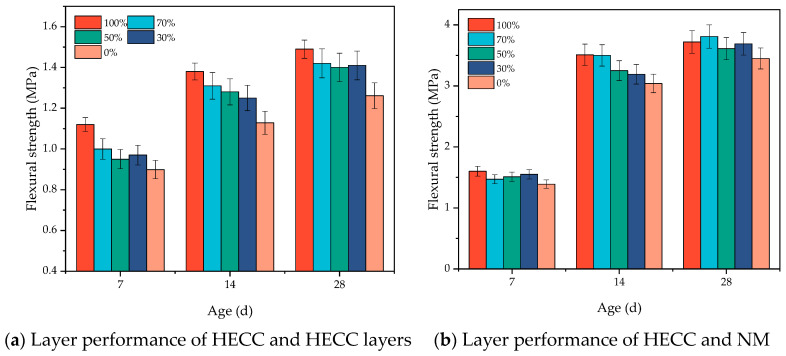
Influence of different degrees of saturation on layer bonding properties.

Combined with the fitting relationship between drying time and saturation degree in the oven in Section 2.3.4, five different saturation degrees were selected, namely 100%, 70%, 50%, 30%, and 0%, which corresponded to durations in the oven of 0 h, 1.6 h, 3.2 h, 5.7 h, and 26.4 h, respectively. The substrates of HECC and NM cured at the age of 28 days (i.e., the interval pouring time was 28 days) were taken and placed in the oven for the time mentioned above so that different saturation degrees could be obtained, and then the other half of the cover layer of the HECC was poured. The results for the HECC and HECC layers are shown in Figure 17a, where it is found that the layer flexural strength of the specimens with 100% substrate saturation is the highest and that of the specimens with 0% saturation is the lowest. The rest of the groups show nearly similar flexural strengths, and the layer flexural strengths of the specimens that are completely saturated are 18% higher than those of the specimens that are completely dry. For the HECC and NM layer results shown in Figure 17b, the difference between the different saturations is smaller, with the completely dry specimens having the lowest layer flexural strength, which is about 15% lower than the remaining groups.

The results of most of the experiments show that a moist substrate provides higher bond strength than a dry substrate, with the main reason being that a dry interface causes incomplete hydration of the cement near the layer, generating a large number of air bubbles, creating weak areas, and reducing bond strength [20]. Bentz et al. [40] used pullout tests to obtain the same conclusion as the present experiments and found that a moist substrate provides a higher bond strength. Farzad et al. [22] similarly found that a moist substrate performed better in double-sided shear and bending tests, providing higher bond strength under all tests. However, for the differences between different saturations, the results of the current studies vary considerably. Beushausen et al. [26] investigated the effect of different saturations, but the data varied considerably. Also, the use of different test methods produces different results, with flexural and tensile tests often revealing a higher strength of the moist substrate, with the opposite conclusion in diagonal shear experiments [40]. A deeper understanding of the micromechanics at the interface and the principles of fluid–solid interaction can help to further investigate the differences in the strength of the layers due to different saturation layers.

#### 3.2.4. Effect of Surface Roughness on Layer Performance

**Figure 18:**
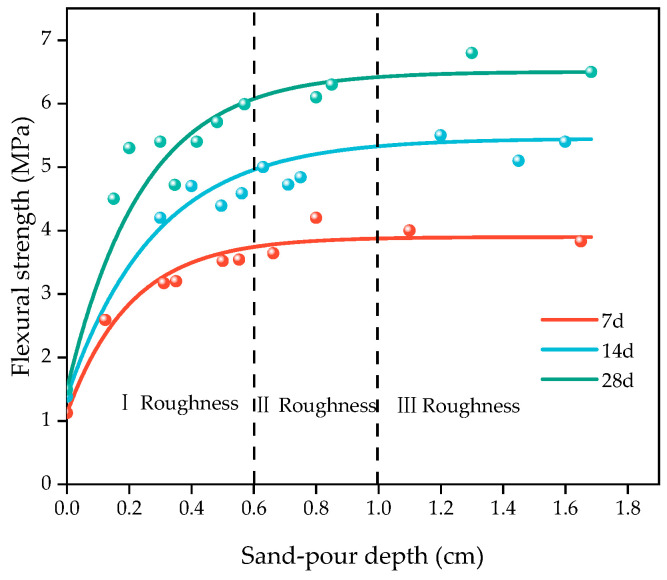
The variation law of HECC and HECC layer flexural strength with sand-pour depth.

**Figure 19:**
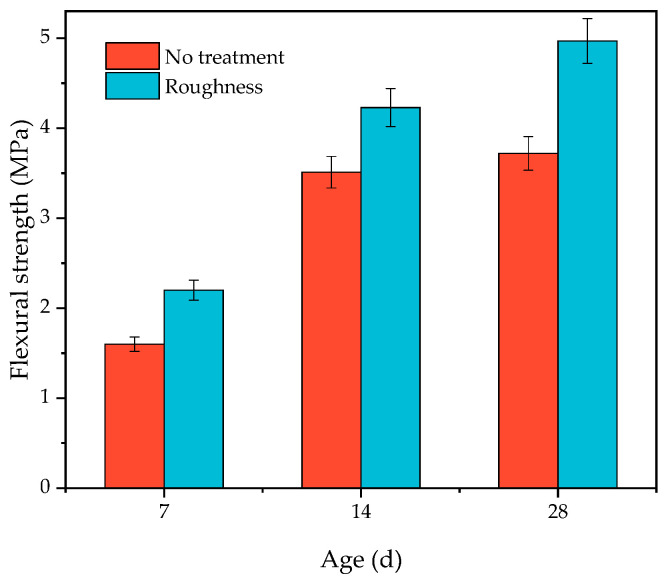
The variation law of HECC and NM layer flexural strength with sand-pour depth.

In accordance with the method described in Section 2.3.5, the substrate of the HECC and NM maintained for 28 days of age (i.e., the interval pouring time was 28 days) was taken, the surface sand-pour depth was measured, and then the other half of the overlayer, HECC, was poured. Figure 18 demonstrates the trend of the sand-filling depth and the flexural strength of the layer after HECC and HECC layer specimens were cured at different ages, and the results show that the improvement in the flexural strength of the layer was the greatest for Type I roughness, with only a small improvement of about 180–200%; the improvement of Type II roughness was only about 5–10%; and Type III roughness had almost no improvement. Figure 19 shows the improvement of NM roughness on the flexural strength of the surface, and the roughness of the surface could make the flexural strength of the surface increase by 20.5–37.5%. (In the figure, no treatment represents an untreated flat surface, and roughness represents a surface treated according to the Section 2.3.5 method.)

Surface roughness results in a larger contact area between the substrate and the overlay, leading to better layer bonding. As the roughness increases, the bond strength increases, but the roughness cannot be increased indefinitely without causing damage to the substrate surface; at the same time, a lower roughness can provide greater layer bond strength [41]. Hu et al. [41] used the split tensile and shear test to find that the strength improvement is greater at lower and intermediate layers of the depth of the sand filling and that the strength does not improve or even decrease for higher layers. Zhang et al. [42] and Momayez et al. [43] also found that a rough surface has a positive effect on the layer. However, different methods of creating roughness have slightly different enhancement effects, and traditional methods such as chiseling and sandblasting enhance the bond strength of the layers by 20–90% in split tensile and shear tests [44].

## 4. Conclusions

This paper explored the factors that impact the layer bonding properties of hydraulic engineered cementitious composites. These properties were evaluated through measurements of layer compressive strength and layer flexural strength. The findings can be summarized as follows:

1. The layer bond strength exhibited a noticeable decline as the pouring interval increased. It is noteworthy that a layer formed only when a pouring interval was present. And the bond strength of concrete layers was lower than 50% of concrete with an uninterrupted pouring and molding process. Pouring intervals of 2.5 h and 7 days or longer tended to be relatively similar.
2. The horizontal flexural strength exceeded the vertical flexural strength, while the horizontal compressive strength was lower than the vertical compressive strength. Moreover, with an increase in pouring interval time, there was a trend of initially decreasing and then increasing strength.
3. The layer bonding properties of the HECC and HECC 100% saturation substrate layer exhibited flexural strength close to the highest, and that of the 0% saturation substrate was the lowest. As for the layer bonding properties of HECC and NM, different degrees of saturation of the substrate resulted in similar layer flexural strengths.
4. The HECC substrate had a significant layer flexural strength enhancement of nearly 200% at Class I roughness. The increase became less pronounced at Class II roughness and was almost negligible at Class III. The surface roughness of the NM substrate led to a layer flexural strength enhancement of 20.5–37.5%.

The influencing factors of the bonding properties of HECC and HECC layers and HECC and NM layers were investigated, but the study is limited to the macroscopic mechanical behavior, and further research on the microstructure of the layers is necessary to elucidate the underlying mechanism.

## Figures and Tables

**Table 1:** Basic properties of cement.

Type	Blain Area	Setting Time		Compressive Strength		Flexural Strength	
	(m^2^/kg)	Initial Setting Time (min)	Final Setting Time (h:min)	(MPa)		(MPa)	
				3 Days	28 Days	3 Days	28 Days
Esheng42.5	334	181	4:19	27.5	47.4	6.3	8.6
GB175-2020	≥300	≥45	≤10:00	≥17.0	≥42.5	≥3.5	≥6.5

**Table 2:** Basic properties of fly ash.

Type	Fineness (%)	Blain Area	Moisture Content (%)	Ratio of Water Requirements (%)	Compressive Strength Ratio (%)		Ignition Loss (%)
		(m^2^/kg)			7 Days	28 Days	
Jintang	6.8	390	0.1	95	68	75	2.8
DL/T5055-2007	≤12.0	–	≤1.0	≤95	–	–	≤5.0

**Table 3:** Chemical compositions of cement and fly ash (wt.%).

Oxide	Cement	Fly Ash
SiO_2_	21.41	48.33
Al_2_O_3_	4.95	17.58
Fe_2_O_3_	3.81	8.63
CaO	59.36	8.73
MgO	0.94	2.89
K_2_O	0.75	1.41
Na_2_O	0.13	0.75
SO_3_	3.11	1.86
LOI	2.59	3.44
Na_2_O_eq_	0.62	1.68

**Table 4:** Quality indicators of fiber.

Type	Diameter (μm)	Length (mm)	Density (g/cm)	Breaking Strength (MPa)	Elastic Modulus (GPa)	Fracture Elongation (%)
Wanwei	37	12	1.3	1800	34	6.6

**Table 5:** Artificial sand particle distribution (%).

Particle Size Range	1.25~0.63	0.63~0.32	0.32~0.16	<0.16
Distribution	25.5	22.1	20.6	19.2

**Table 6:** Test results of concrete properties of SP tested.

Type		SP	DL/T 5100-2014
Dosage (%)		0.8	\
Water-reducing rate (%)		26.9	≥25
Gas content (%)		2.3	≤2.5
Bleeding rate ratio (%)		30	≤60
Setting time difference (min)	Initial setting time	+152	≥90
	Final setting time	+132	\
Compressive strength ratio (%)	3 days	145	\
	7 days	140	≥140
	28 days	137	≥130
Shrinkage ratio (%)		97	≤110

**Table 7:** The mixture proportions of HECC and NM.

Type	Cement	Fly Ash	Artificial Sand	Water	SP	VMA	PVA Fiber (Volume/%)
HECC	1	1	1.5	0.66	0.016	0.0001	2
NM	1	1	2.5	0.66	0.016	\	\

**Table 8:** Pouring interval number and corresponding meaning.

Number	Meaning
D0	No pouring interval time
D1	20 min pouring interval time
D2	40 min pouring interval time
D3	60 min pouring interval time
D4	2.5 h pouring interval time
D5	7-day pouring interval time
D6	14-day pouring interval time
D7	28-day pouring interval time

## Data Availability

Not Applicable.
